# Parallel SPR and QCM-D Quantitative Analysis of CD9, CD63, and CD81 Tetraspanins: A Simple and Sensitive Way to Determine the Concentration of Extracellular Vesicles Isolated from Human Lung Cancer Cells

**DOI:** 10.1021/acs.analchem.3c00772

**Published:** 2023-06-12

**Authors:** Agata Kowalczyk, Aleksandra Gajda-Walczak, Monika Ruzycka-Ayoush, Alicja Targonska, Grazyna Mosieniak, Maciej Glogowski, Anna Szumera-Cieckiewicz, Monika Prochorec-Sobieszek, Magdalena Bamburowicz-Klimkowska, Anna M. Nowicka, Ireneusz P. Grudzinski

**Affiliations:** † Department of Inorganic and Analytical Chemistry, Faculty of Chemistry, University of Warsaw, Pasteura Street 1, PL-02-093 Warsaw, Poland; ‡ Department of Toxicology and Food Science, Faculty of Pharmacy, Medical University of Warsaw, Banacha Streer 1, PL-02-097 Warsaw, Poland; § Laboratory of Molecular Bases of Ageing, Nencki Institute of Experimental Biology, Polish Academy of Sciences, Pasteura Street 3, PL-02-093 Warsaw, Poland; ∥ Department of Lung Cancer and Chest Tumors, Maria Sklodowska-Curie National Research Institute of Oncology, Roentgena Street 5, PL-02-781 Warsaw, Poland; ⊥ Department of Cancer Pathomorphology, Maria Sklodowska-Curie National Research Institute of Oncology, Roentgena Street 5, PL-02-781 Warsaw, Poland

## Abstract

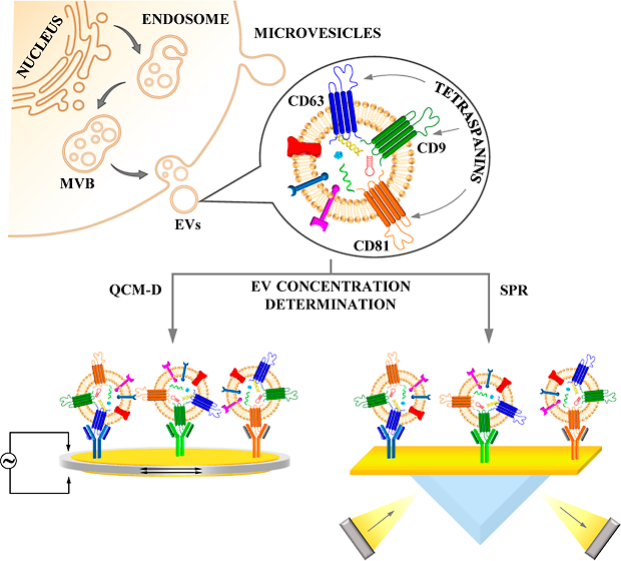

Tetraspanins, including CD9, CD63, and CD81, are transmembrane biomarkers that play a crucial role in regulating cancer cell proliferation, invasion, and metastasis, as well as plasma membrane dynamics and protein trafficking. In this study, we developed simple, fast, and sensitive immunosensors to determine the concentration of extracellular vesicles (EVs) isolated from human lung cancer cells using tetraspanins as biomarkers. We employed surface plasmon resonance (SPR) and quartz crystal microbalance with dissipation (QCM-D) as detectors. The monoclonal antibodies targeting CD9, CD63, and CD81 were oriented vertically in the receptor layer using either a protein A sensor chip (SPR) or a cysteamine layer that modified the gold crystal (QCM-D) without the use of amplifiers. The SPR studies demonstrated that the interaction of EVs with antibodies could be described by the two-state reaction model. Furthermore, the EVs’ affinity to monoclonal antibodies against tetraspanins decreased in the following order: CD9, CD63, and CD81, as confirmed by the QCM-D studies. The results indicated that the developed immunosensors were characterized by high stability, a wide analytical range from 6.1 × 10^4^ particles·mL^–1^ to 6.1 × 10^7^ particles·mL^–1^, and a low detection limit (0.6–1.8) × 10^4^ particles·mL^–1^. A very good agreement between the results obtained using the SPR and QCM-D detectors and nanoparticle tracking analysis demonstrated that the developed immunosensors could be successfully applied to clinical samples.

## Introduction

Cell-to-cell communication is essential for proper function in multicellular organisms, and extracellular vesicles (EVs) play a critical role in this process. Cells actively secrete EVs specifically targeted to other cells to convey complex information in this communication.^1^ Because of their unique composition and functions, EVs have opened up the possibility of their practical use in the diagnosis of many diseases, making them a subject of intense interest within the scientific community. In recent years, much attention has been focused on their role in lung cancer, which is one of the most common malignant tumors worldwide and remains a significant cause of cancer incidence and mortality.^2^ In the early stages of lung cancer, symptoms may not be obvious and can be easily overlooked.

EVs are nanometer-sized biological structures that are released from almost all cells under physiological and pathological conditions. These vesicles contain many active molecules, such as proteins, lipids, and genetic material in the form of different RNA and DNA species.^3^ The diameter of EVs largely depends on their origin and typically ranges from 30 to 200 nm.^4^ The quantification of EVs in a solution is mainly based on methods such as electron microscopy (EM),^5,6^ nanoparticle tracking analysis (NTA),^7,8^ dynamic light scattering (DLS),^9^ and ELISA tests.^10^ However, these methods work best with impurity-free solutions, and the presence of other types of membrane microbubbles in the analyzed solution in sizes similar to EVs significantly hinders NTA and DLS measurements.^11^ In contrast, EM allows the distinction between EVs and other extracellular vesicle-type particles. Unfortunately, EVs are counted manually by the operator, using this technique, making it very tedious and time-consuming and requiring qualified staff. Moreover, during the sample preparation step, many EVs are damaged, leading to an underestimation of their actual number by EM.^12^

The quantitative analysis focused on the origin of EVs can be an effective diagnostic tool. The substances contained inside EVs, as well as those that are part of the membrane, are strongly affected by the disease state. Characteristic components of the EV membrane include molecules such as transport and fusion proteins (GTPases, annexins, and flotillin), tetraspanins (CD9, CD63, CD81, and CD82), heat shock proteins (Hsc70 and Hsp90), proteins involved in MVB formation [Alix (ALG-2-interacting protein X)], TSG101 (tumor susceptibility gene 101), as well as lipid-related proteins and phospholipases.^13−18^ In the context of EVs as biomarkers for lung cancer, attention is mainly focused on the EV-derived miRNAs and proteins such as PD-L1, EGFR, and TTF-1.^19^ Furthermore, proteins such as CD151, TSPAN8, and CD171 are involved in the progression of lung carcinogenesis and are highly expressed in the EVs of lung cancer patients relative to noncancer patients.^20,21^ Commercial quantitative analysis of EVs as cancer biomarkers is mainly based on ELISA tests, western blotting, or PCR. However, these methods have some drawbacks, such as being complicated, time-consuming, cost-consuming, and requiring high sample volumes. Meanwhile, other techniques such as fluorescence^22^ or flow cytometry^23^ require prior labeling of EVs with an appropriate tag.

The low concentration of tumor cell-derived EVs in body fluids and the limited variety of characteristic components make their quantitative analysis challenging. However, this challenge has been addressed by combining information about the kinetics of EVs’ attachment to a surface plasmon resonance (SPR) sensor and the changes in mass and viscoelastic properties that occur when EVs adhere to the surfaces of piezoelectric quartz sensors.

In SPR, a ligand such as an antibody, enzyme, peptide, or DNA is immobilized on the chip surface to capture the analyte from the sample solution flowing across the SPR surface. The formation of the ligand–analyte complex leads to changes in the refractive index. Since the dimension of EVs closely matches the evanescent wave that propagates for about 200 nm from the chip surface,^24^ SPR provides an effective way for label-free and rapid EV detection.

Quartz crystal microbalance with energy dissipation (QCM-D) is a label-free, extremely sensitive mass balance technique. The basic element of this technology is a quartz disc, a piezoelectric material that can be made to oscillate at a defined frequency by applying an appropriate voltage.^25^ The changes in frequency, measured in real-time, are the consequence of the addition or removal of molecules from the quartz crystal during the interaction process. QCM-D allows for data acquisition at multiple overtones of a crystal’s fundamental resonance frequency. The low overtone number reflects the process in the depth of the solution, while the high overtone number reflects the process directly near the crystal surface.^26,27^

In this study, we report on the development of amplifier-free SPR and QCM-D immunosensors for the direct and sensitive quantification of lung cancer cell-derived EVs. The detection process was based on antigen–antibody interaction, and we focused on the three tetraspanins, CD9, CD63, and CD81. CD9 is broadly expressed in non-small cell lung cancer (NSCLC) lines but is absent or highly reduced in most small cell lung cancer (SCLC) lines, while CD63 and CD81 are broadly expressed in both SCLC and NSCLC lines.^28^ Antibody molecules, specific to selected tetraspanins, were introduced to the receptor layer in an orientation consistent with vertical orientation through protein A (SPR detection) or a cysteamine layer (QCM-D detection). The simultaneous determination of these three tetraspanins present in the membrane of EVs can be applied to lung cancer diagnosis. Until this moment, QCM-D and SPR studies on lung cancer cell-derived EVs focused mainly on CD63 detection.^29−31^ The proposed protocol was validated for its specificity, limit of detection (LOD), and limit of quantification (LOQ). The proposed EV assay protocols were also validated against clinical samples.

## Materials and Methods

### Materials

Cysteamine hydrochloride (CSH), *N*-hydroxysuccinimide (NHS), and *N*-(3-dimethylaminopropyl)-*N*′-ethylcarbodiimide hydrochloride (EDC) were purchased from Merck and used as received. Purified anti-human CD9 monoclonal antibody (anti-CD9) and purified anti-human CD81 monoclonal antibody (anti-CD81) were purchased from BioLegend, and a recombinant monoclonal human CD63 antibody (anti-CD63) was purchased from Bio-Techne. All experiments and solutions were conducted in 0.01 M PBS-Gibco (pH 7.4; Thermo Fisher).

### Cell Culture

We obtained the adenocarcinomic human alveolar basal epithelial cell line A549 (ATCC CCL-185) from the American Type Culture Collection (ATCC, Manassas, VA, USA). The cells were cultivated in a CO_2_ incubator (Memmert, Schwabach, Germany) under a 5% CO_2_ atmosphere at 37 °C. They were grown as an adherent monolayer in F-12K Medium (Kaighn’s Modification of Ham’s F-12 Medium; Gibco, Paisley, UK) supplemented with 10% fetal bovine serum (FBS; Gibco, Paisley, UK) and antibiotics (streptomycin, 50 μg·mL^–1^; amphotericin B, 1.25 μg·mL^–1^; gentamicin, 50 μg·mL^–1^; and penicillin, 50 U·mL^–1^) (Gibco, Paisley, UK). Before EV isolation, we replaced the standard media with 10% EVs-depleted FBS media (One Shot format, Gibco, Paisley, UK), and A549 cells were incubated for an additional 3 days in T225 culturing flasks.

### Primary Cell Culture

We derived human primary lung cancer cells from patients who underwent surgical removal of primary lung cancer. The clinical characteristics of the lung tumors are summarized in Table 1. The project was approved by the local Bioethical Committee, and we obtained written informed consent from all patients. To date, the lung cancer specimens were processed immediately after resection. We fragmented the lung cancer tissues using sterile surgical blades into approximately 1 × 1 mm pieces. We then minced the fragments on a grid and seeded them on a plate in F-12K Medium. After establishing the primary cell culture, we transferred the cells to T75 culture flasks (Gibco). Prior to EV isolation, we replaced the standard media with 10% EVs-depleted FBS media (One Shot format, Gibco, Paisley, UK), and we incubated the primary cells for an additional 3 days in T225 culture flasks (Gibco).

**Table 1:** Primary Cancer Cell Line and Size of Lung Cancer Cell-Derived EVs

patient	type of cancer	EVs size [nm]^a^
987	lung squamous cell carcinoma	118.3 ± 1.1
3486	lung adenocarcinoma	123.5 ± 0.6
3994	lung squamous cell carcinoma	116.1 ± 0.8
9303	lung adenocarcinoma	125.0 ± 0.7
cell line A549		98.3 ± 1.2

a NTA.

### Extracellular Vesicle Isolation

We harvested the cell culture media from the primary cell lines and A549 cells and centrifuged it at 750×*g* at 4 °C for 15 min to remove the detached cells. Then, we centrifuged the collected media at 2000×*g* at 4 °C for 20 min to remove the microvesicles. We collected the supernatant and filtered it through 0.45 μm filters. We subsequently spun it in a Beckman Coulter Optima L-80XP ultracentrifuge at 10,000×*g* at 4 °C for 45 min with a Type SW 32 Ti rotor to remove the apoptotic bodies and cell debris. We recovered the supernatant and filtered it through 0.22 μm filters. We then ultracentrifuged it at 100,000×*g* at 4 °C for 90 min to pellet the EVs. We carefully removed the supernatant and resuspended the crude EV-containing pellets in an aliquot of PBS, which we pooled.

### EV Identification

The EVs used in this study were characterized in our recently published paper using various methods and techniques, including transmission electron microscopy with energy-dispersive spectroscopy Super-X windowless drift detectors, Western blot analysis for canonical markers (CD63 and CD81) and tumor susceptibility gene 101 (TSG101), and quantitative protein levels (measured using the BCA assay).^32^

### Nanoparticle Tracking Analysis

We analyzed the mean size and concentration of EVs using a NanoSight NS300 (Malvern Panalytical Ltd., UK) equipped with a 488 nm blue laser. For each measurement, we captured five 30 s videos and analyzed them using the built-in NanoSight Software NTA 3.2. Prior to measurement, we diluted each sample 1:4 in PBS-Gibco.

### Surface Plasmon Resonance

We conducted the SPR experiments using a Biacore X100 from Cytiva. We used a protein A sensor chip (Cytiva) that provided high capture capacity on the Fc region of all human IgG. The measurements were carried out at a flow rate of 5 μL·min^–1^. To ensure reproducibility and standardization, we fixed the parameter values as follows: (i) surface modification of the biochip with antibodies Ab CD9, Ab CD63, and Ab CD81 (contact time: 360 s; stabilization: 300 s; and flow rate: 5 μL·min^–1^) and (ii) EV interactions with selected antibodies (contact time: 90 s; dissociation time: 300 s; and flow rate: 5 μL·min^–1^). The same sensor chip could be used for several experiments, provided that the surface was properly regenerated by removing EVs and captured antibodies. We adopted a two-step regeneration protocol using 10 mM glycine-HCl pH 1.5 and 50 mM NaOH regeneration solutions. These solutions were injected at a flow rate of 30 μL·min^–1^ for 30 min, followed by a stabilization period of 1 min.

### Quartz Crystal Microbalance with Dissipation

The experiments were conducted using a QCM-D E4 instrument (Q-sense AB, Sweden), which was fitted with gold-coated quartz crystals (type QSX 301) that had a frequency of 4.95 MHz. The gold crystals used had a surface roughness of <1 nm RMS and a sputtered gold layer that was approximately 100 nm thick. Before conducting the experiments, the Au crystals were cleaned in a TL1 mixture comprising ultrapure water, 25% ammonia, and 30% hydrogen peroxide (v/v ratio 5:1:1) at 75 °C for 5 min. The crystals were then rinsed with ultrapure water, followed by ethanol (99.8%), and dried with an Ar stream. The QCM-D measurements were carried out using a flow system at a flow rate of 100 μL·min^–1^ and a constant temperature of 21 °C. Prior to each solution exchange in the QCM-D chamber, a stable baseline in pure 0.01 M PBS-Gibco (pH 7.4) was obtained. To ensure that the antibody molecules were vertically oriented (maximizing the efficiency of the antigen recognition process) at the surface, the gold QCM-D sensors were modified with the CSH monolayer. The chemisorption of the CSH layer was performed in a 1 mM aqueous solution overnight at room temperature. Unspecifically bound CSH chains were removed from the crystal surface by immersing it several times in ethanol and then in water. Only the step of crystal modification with the CSH layer was performed in the open module system. The sensor surface was then carefully dried with an Ar stream and placed in the E4 chamber. Next, the carboxylic groups of CD antibodies were activated for 30 min with a standard aqueous mixture of EDC/NHS (40 mM/10 mM), and the Ab solution was added to the QCM-D chamber. Such modified sensors (Au/CSH/anti-CDs) were then ready to use.

### Atomic Force Microscopy and Scanning Electron Microscopy

All AFM images were acquired using ScanAsyst Fluid + probes (Bruker) in peak force tapping mode with a nominal spring constant of 0.4 N·m^–1^. Each probe was calibrated using the thermal tuning module before use. The images were obtained in an air atmosphere. For the SEM analysis, a low electron beam energy of 3 kV and a current of 30 pA were employed. Before the examination, each sample was coated with a thin film (1–3 nm) of Au–Pd alloy to prevent electrical charging of the sample surface. The alloy layers were deposited using a Polaron SC7620 Mini Sputter Coater.

## Results and Discussion

Directed covalent binding methods enable the precise orientation of antibody molecules relative to the substrate, ensuring the maximum efficiency of antigen recognition.^33^ Carboxyl groups located at the ends of the polypeptide chains of the constant domain of the antibody (Fc region) are utilized for this purpose, which leads to an orientation consistent with the vertical orientation of Ab molecules relative to the substrate surface. In such a case, the surface of the substrate should be previously modified with a layer containing amino groups. Alternatively, protein bridges can be formed between the antibody and the matrix *via* proteins A, G, or L^34,35^ to target antibody binding. These proteins are small molecules (30–106 kDa) of bacterial origin, such as *Staphylococcus aureus* (protein A),^36^*Streptococcus C40* (protein G),^37^ and *Peptostreptococcus magnus* (protein L),^38^ and they exhibit characteristics of mammalian class G immunoglobulins.^34,39^ Proteins A and G bind to the Fc region of antibody heavy chains, while protein L binds to light chains outside the antigen binding site.

**Figure 1:**
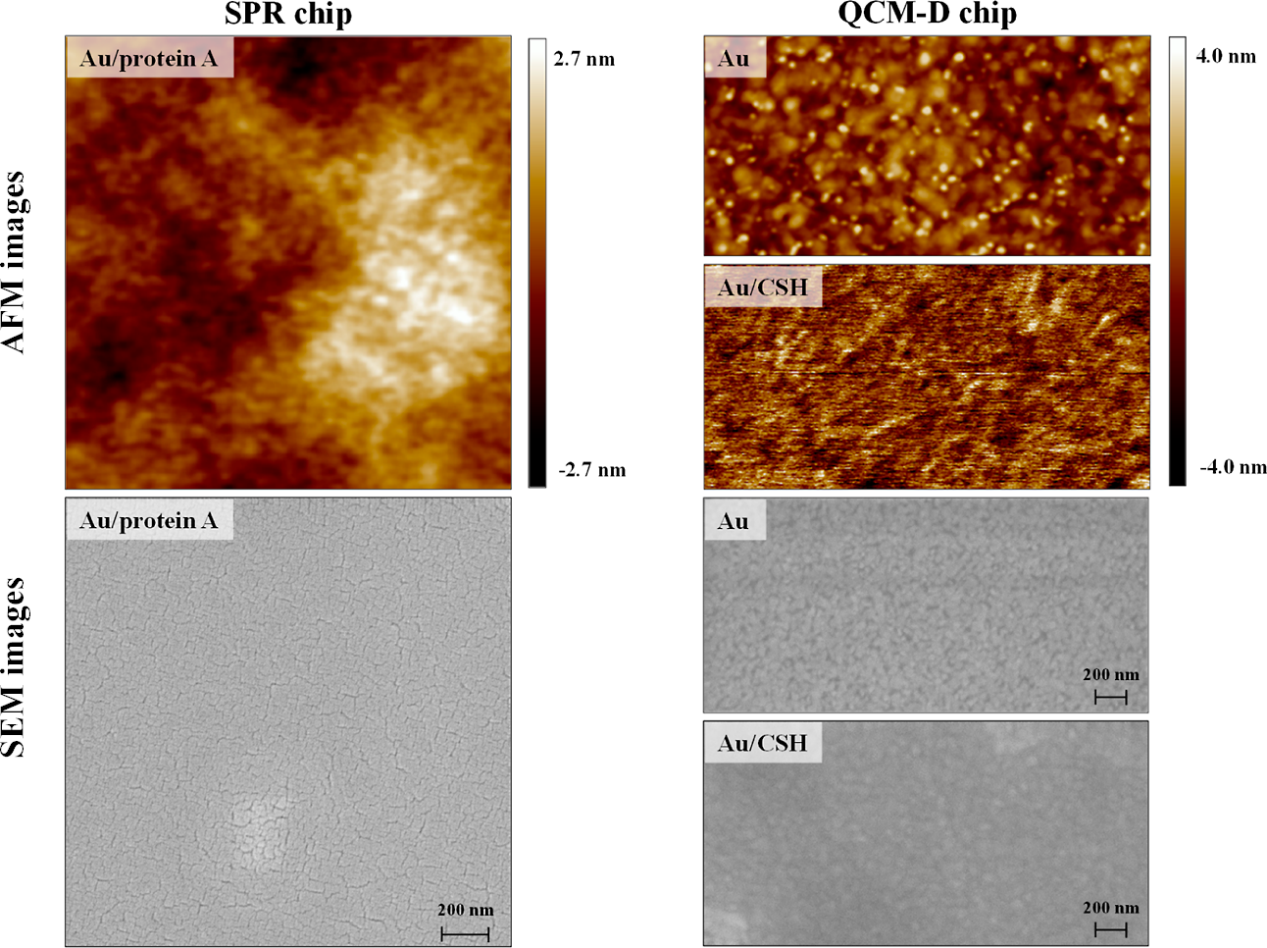
AFM and SEM images of the SPR and QCM-D chip surfaces.

To ensure the maximum efficiency of the antigen–antibody recognition process, which is only guaranteed by the vertical arrangement of Ab molecules in the receptor layer, we utilized a commercially available protein A SPR chip in the SPR studies and gold quartz crystals modified with a cysteamine monolayer in the QCM-D studies. The vertical orientation of the antibody molecules in the receptor layer is determined not only by their method of insertion but also by the degree of roughness of the substrate. Both the substrates used were characterized by a smooth surface, which was confirmed by SEM and AFM analyses presented in Figure 1.

### SPR Analysis

**Figure 2:**
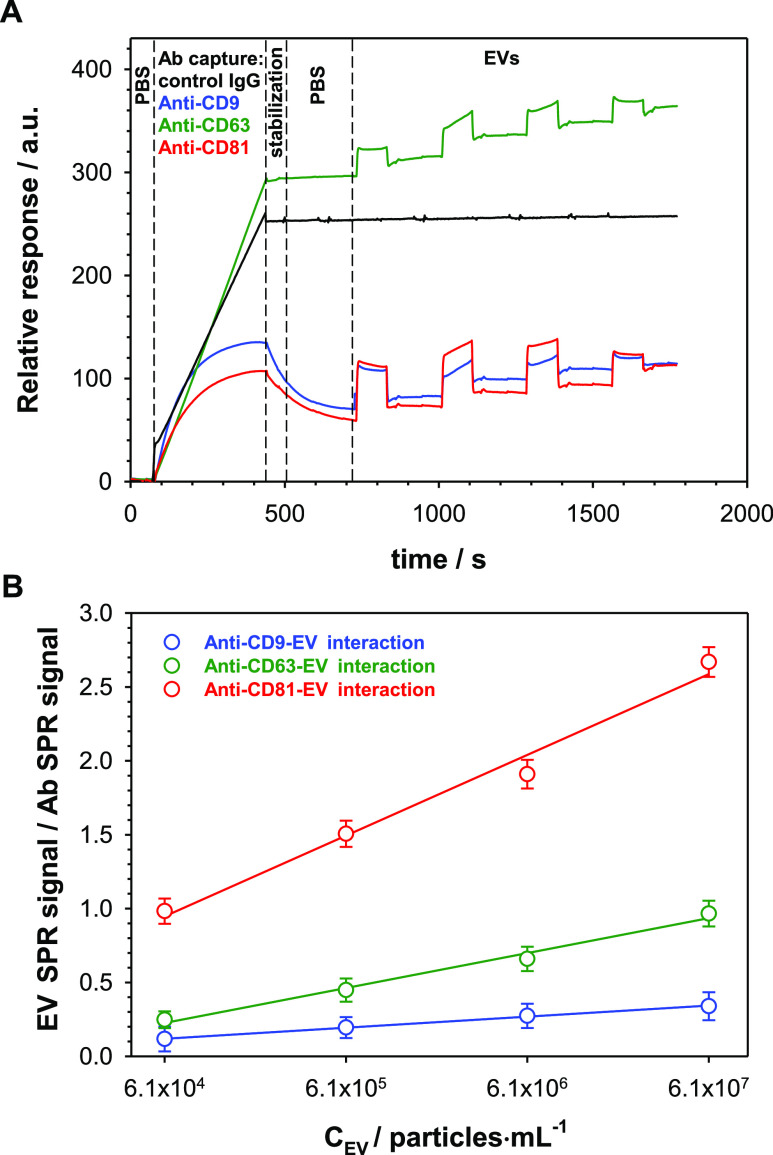
(A) Sensorgrams recorded for the interactions of anti-CD9 (black line), anti-CD63 (green line), and anti-CD81 (red line) interactions with EVs derived from the A549 cell culture medium. (B) Calibration plots. Experimental conditions: 0.01 M PBS-Gibco (pH 7.4; 0.005% Tween), *C*_Abs_ = 1.0 μg·mL^–1^, and *C*_EVs_: 6.1 × 10^4^ to 6.1 × 10^7^ particles·mL^–1^.

The presence of tetraspanins such as CD9, CD63, and CD81 on the surface of EVs isolated from the A549 cell culture medium was confirmed by SPR analysis. Figure 2A shows representative real-time SPR response curves recorded during the binding process of EVs with subsequent antibodies (Ab: anti-CD9, anti-CD63, and anti-CD81).

To estimate the kinetic parameters of the Ab + antigen (Ag) ↔ Ab–Ag reaction, such as the association rate (*k*_a_), dissociation rate (*k*_d_), and equilibrium dissociation constant (*K*_D_), the antibody molecules need to be anchored to the SPR chip *via* their Fc domains. This approach should not sterically hinder Ag binding to the variable region, enabling reliable characterization of Ab–Ag interactions. To achieve the orientation of Ab molecules consistent with their vertical orientation relative to the surface, the SPR protein A sensor chip was utilized. All measurements were repeated at least three times, and the average values of the kinetic parameters are provided in Table 2. The structure of tetraspanins contains four transmembrane alpha-helices and two extracellular domains: one short (small extracellular domain/loop) and one longer (large extracellular domain/loop). Therefore, the best model describing the interactions of tetraspanins present on the EVs with specific monoclonal antibodies is a two-state reaction model
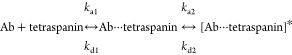
1

**Table 2:** Kinetic Parameters of Interactions between EVs and Anti-CD9, Anti-CD63, and Anti-CD81 Using a Two-State Reaction Model

	anti-CD9	anti-CD63	anti-CD81
*k*_a1_ [M^–1^·s^–1^]	(1.67 ± 0.05) × 10^2^	(3.48 ± 0.02) × 10^3^	(1.76 ± 0.10) × 10^2^
*k*_d1_ [s^–1^]	(6.66 ± 0.12) × 10^–4^	(9.41 ± 0.11) × 10^–1^	(1.43 ± 0.09) × 10^–2^
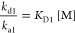	3.99 × 10^–6^	2.70 × 10^–4^	8.13 × 10^–5^
*k*_a2_ [s^–1^]	(7.46 ± 0.07) × 10^–2^	(6.81 ± 0.11) × 10^–2^	(1.67 ± 0.09) × 10^–1^
*k*_d2_ [s^–1^]	(1.87 ± 0.03) × 10^–6^	(1.11 ± 0.05) × 10^–5^	(1.06 ± 0.07) × 10^–2^
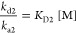	2.51 × 10^–5^	1.63 × 10^–4^	6.35 × 10^–2^
*K*_D_ = *K*_D1_•*K*_D2_ [M]	10.00 × 10^–11^	4.10 × 10^–8^	5.16 × 10^–6^
*K*_D_^a^ [M]	9.98 × 10^–11^	4.41 × 10^–8^	4.77 × 10^–6^

a Value determined by fitting a two-state reaction model.

In the selected model, each reaction state is described by an individual equilibrium dissociation constant, and the total equilibrium dissociation constant is the product of the stepwise equilibrium constants. The chosen model correctly describes the interactions of EVs with selected monoclonal tetraspanin antibodies since the dependencies ln(*R*_0_/*R*) = *f*(*t*), where *R*_0_ is the response level at the beginning of the post-injection phase for each EV concentration, presented in Figure S1 in the Supporting Information, are linear.^40^

The performed kinetic analysis showed the existence of two equilibrium dissociation constants. In the first step, EVs bind relatively weakly to the antibody, followed by a conformational change in the epitope structure (the most flexible part of the antigen), leading to a more stable complex between the antibody and antigen (low total *K*_D_). Similar behavior was also observed by others.^41−43^ When considering the total *K*_D_ value (last row in Table 2), it is evident that the EVs produced by adenocarcinoma human alveolar basal epithelial cells exhibited the highest binding affinity to anti-CD9 and the weakest to anti-CD81, which is a consequence of the distribution of the relevant receptors in the cell membrane of EVs. However, for all studied antibodies, the total *K*_D_ values were lower than 0.1 μM, indicating high binding affinity.^44^ The differences in binding affinity are due to the differences in association and dissociation rates. It should be emphasized that the interactions between the selected EVs membrane proteins of the tetraspanin family and the antibodies are characterized by similar association rates and completely different dissociation rates. Association rates for antigen–antibody interactions cover a wide range, from approximately 10^2^ to 10^9^ M^–1^·s^–1^, with most cases in the order of 10^5^ to 10^6^ M^–1^·s^–1^.^45^ Meanwhile, dissociation rates for this type of interaction range from 10^–6^ to 10^–1^ s^–1^, with most cases in the order of 10^–6^ to 10^–4^ s^–1^.^45^ Faster association rates are a consequence of the electrostatic type of interaction,^46^ while slower association rates result from processes such as desolvation and structural rearrangements.^47,48^ Given the complexity of EVs membrane receptors, for the interaction of the selected receptor (tetraspanin) with the antibody to occur, a structural rearrangement of the receptors is necessary. Moreover, for all analyzed interactions, the *K*_D_ values obtained by fitting the two-state reaction model are in general agreement with the ratios of the corresponding kinetic parameters (*k*_d_/*k*_a_).

To investigate the analytical parameters, including dynamic concentration range, sensitivity, and LOD and LOQ, designated volumes of EVs were spiked in PBS-Gibco to obtain desired dilutions: 6.1 × 10^4^; 6.1 × 10^5^; 6.1 × 10^6^; and 6.1 × 10^7^ particles·mL^–1^ and driven through the SPR sensor chip (Au-protein A/Ab). The initial concentration of isolated EVs derived from lung cancer cells (A549 cell line) was specified from NTA measurements and equaled 6.1 × 10^8^ particles·mL^–1^. Figure 2B presents the changes in the ratio of EVs SPR signal/Ab SPR signal *versus* EV concentration. For all studied antibodies, the dependencies were linear, and the regression equations describing them are given in Table 3. The LODs for each tetraspanin antibody were estimated by taking into account the variability of the background SPR responses measured in the pure PBS-Gibco buffer solution. The LOD values were determined as three standard deviations of the five controls. For all applied EV concentrations, the repeatability was satisfactory, with the relative standard deviation being approximately 9.8% (*n* = 3). The chip-to-chip reproducibility was examined for three different chips on the steps of (i) antibody capture and (ii) antibody EV interactions, and the relative standard deviation was found to be (i) 5.4, 3.8, and 6.2% for anti-CD9, anti-CD63, and anti-CD81, respectively, and (ii) 7.1, 8.3, and 9.5% for EV interactions with anti-CD9, anti-CD63, and anti-CD81, respectively. The LOQs were calculated as 3.3× LOD. The estimated LOD and LOQ values are presented in Table 3.

**Table 3:** Values of the Analytical Parameters Estimated for SPR and QCM-D Biosensors

	linear regression equation	*R*2	dynamic range [particles·mL^–1^]	LOD/LOQ [particles·mL^–1^]
SPR analysis				
anti-CD9	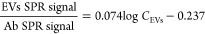	0.9988	6.1 × 10^4^ to 6.1 × 10^7^	7.8 × 10^3^/2.6 × 10^4^
anti-CD63	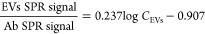	0.9861	6.1 × 10^4^ to 6.1 × 10^7^	0.95 × 10^4^/3.1 × 10^4^
anti-CD81	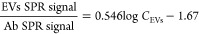	0.9900	6.1 × 10^4^ to 6.1 × 10^7^	2.5 × 10^4^/8.3 × 10^4^
QCM-D analysis				
anti-CD9	Δ*f* = 1.75 log *C*_EVs_ – 6.03	0.9991	6.1 × 10^4^ to 6.1 × 10^7^	0.60 × 10^4^/2.0 × 10^4^
anti-CD63	Δ*f* = 2.02 log *C*_EVs_ – 6.50	0.9993	6.1 × 10^4^ to 6.1 × 10^7^	1.8 × 10^4^/5.9 × 10^4^
anti-CD81	Δ*f* = 1.30 log *C*_EVs_ – 4.07	0.9895	6.1 × 10^4^ to 6.1 × 10^7^	0.70 × 10^4^/2.3 × 10^4^

To confirm that EVs bind to the chip surface only through interaction with antibodies specific to selected tetraspanins, an experiment was performed using control IgG. On the SPR sensorgram (black curve in Figure 2A), a negligible background signal was observed during the detection steps, indicating that the developed protocol is specific in capturing EVs from the samples.

### QCM-D Analysis

**Figure 3:**
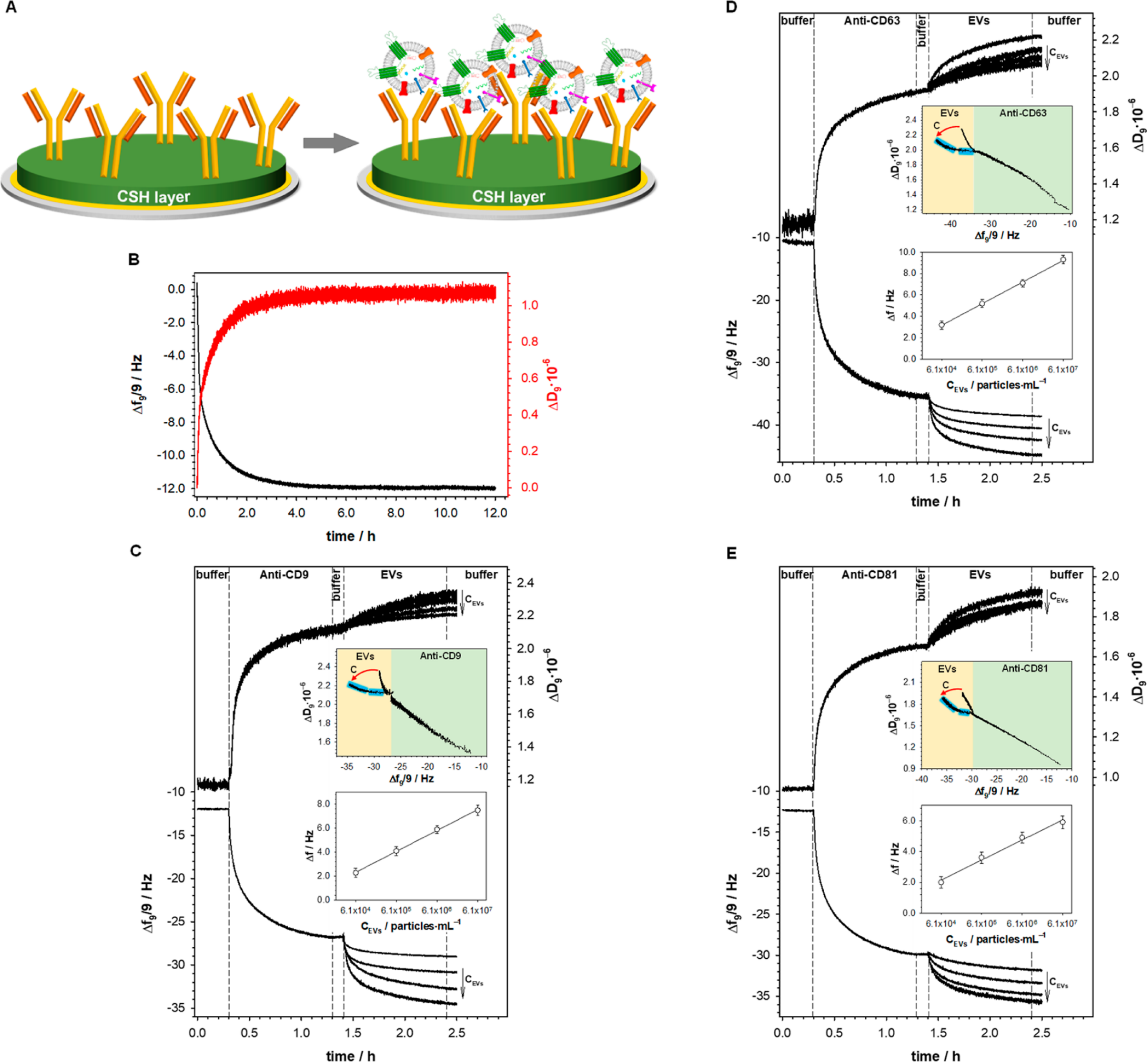
(A) Scheme of gold crystal modification. (B–E) Typical frequency (Δ*f*) and dissipation factor (Δ*D*) changes recorded during the modification of the gold substrate with a cysteamine layer (B), appropriate antibody immobilization, and after its interaction with EVs (C–E). Top insets: dependencies of Δ*D* vs Δ*f* of antibody covalent anchoring to the modified CSH layer Au crystal surface (green area of the plots) and Abs interaction with EVs in two extreme values of concentration (yellow area of the plots; the arrow shows the direction of concentration increase). Bottom insets: calibration plots. Experimental conditions: 0.01 M PBS-Gibco (pH 7.4), *C*_Abs_ = 1.0 μg·mL^–1^, and *C*_EVs_: 6.1 × 10^4^ to 6.1 × 10^7^ particles·mL^–1^.

To eliminate random antibody orientation in the sensing layer, the antibody molecules were anchored to the gold crystal surface through the cysteamine layer. Cysteamine contains amine groups which are directed toward the solution after the chemisorption of CSH chains on the gold surface. The Ab molecules were introduced to the sensing layer using the amide bond formed between the −NH_2_ groups of the thiol and the −COOH groups of the Fc fragment of the antibody.^33^ This method of antibody immobilization should guarantee the vertical orientation of the antibody in the formed layer, ensuring the highest efficiency of antigen–antibody interaction, as seen in the scheme in Figure 3A. It should be stressed that due to the strong adsorption of the thiols on the metallic surface,^49,50^ the modification of the QCM-D Au sensor was performed in the open module chamber (under stationary conditions). The frequency shift (Δ*f*) related to the self-assembly process of CSH, shown in Figure 3B, was approximately 12 Hz, which gives the CSH surface concentration value equal to 2.75 nmol·cm^–2^ (Γ_CSH_ = Δ*f*·*C*_f_/*M*_CSH_, where *C*_f_ is the mass sensitivity of the applied quartz crystal microbalance (17.7 ng·cm^–2^) and *M*_CSH_ is the molecular weight of CSH). Next, the CSH-modified Au crystal was placed in the flow QCM-D chamber, and after reaching a stable baseline in pure 0.01 M PBS-Gibco (pH 7.4), the adsorption process of Ab molecules was initiated by exchanging the buffer with the enzyme solution (1.0 μg·mL^–1^ in 0.01 M PBS-Gibco, pH 7.4). After at least 60 min, the solution was changed back to the buffer (0.01 M PBS-Gibco) to remove the unbound antibody molecules. The addition of appropriate anti-CD to the QCM-D chamber resulted in a further decrease in frequency to about −27, −37.5, and −30.3 Hz for anti-CD9, anti-CD63, and anti-CD81, respectively, as seen in Figure 3C–E. This decrease was a consequence of the covalent bonding of Ab molecules to the amine groups of the CSH layer. After about an hour, the Δ*f* reached a stable value, indicating that the maximal amount of Ab molecules was bound to the CSH layer. To ensure that the applied Ab concentration (1.0 μg·mL^–1^) was enough to fully saturate the layer, control experiments using two different Ab concentrations (two times lower and two times higher than 1.0 μg·mL^–1^) were performed. The differences in the frequency and dissipation intensities and shapes for all applied Ab concentrations were negligible, indicating that the selected Ab concentration for the experiments was correct. QCM-D measures the so-called “wet protein mass”, which is about 30% higher than the “dry protein mass” (stripped of its hydration shell).^51^ Based on the frequency shift (less the contribution of the hydration shell) characteristic of the formation of each antibody layer, the surface concentrations of Ab estimated in the same manner as in the case of CSH were equal to 7.75, 6.09, and 8.68 pmol·cm^–2^ for anti-CD9, anti-CD63, and anti-CD81, respectively. The theoretical Ab surface concentration was evaluated assuming a closely packed 2D structure and the dimensions of antibodies of type IgG (Fab arms: 6.5 × 3.5 nm and Fc stem: 5 × 3.5 nm).^52^ The resulting values were 7.30, 4.52, and 2.43 pmol·cm^–2^ for vertical, tilted, and horizontal Ab orientations, respectively. By comparing these values with those obtained during the Ab immobilization step, it was proven that the applied method of Ab anchoring resulted in the orientation most consistent with the vertical orientation of Ab molecules in the layer.

The determined Ab surface concentrations suggest that the anti-CD9 and anti-CD81 layers are more regular than the layer formed by anti-CD63. This observation was confirmed by comparing the slopes of the dependence Δ*D* = f(Δ*f*) for the steps of Ab layer formation. Plotting the dependence Δ*D* = f(Δ*f*) provides information about the layer organization, mechanical properties, and the amount of dissipation caused by a frequency input. A smaller slope value of this dependence indicates the rigidity of the formed layer. As the value of the slope increases, the layer becomes more viscoelastic.^53,54^ After introducing EVs to the surface of the quartz crystal modified with antibodies, the mechanical properties of the layer changed significantly, as evidenced by the change in the slope of the relationship. The lowest slope value was observed for anti-CD63. It should be noted that the differences in slopes were slight and ranged from −0.029 to −0.043. Moreover, the Δ*D versus* Δ*f* dependence plotted for the step of antibody covalent attachment to the CSH layer scales linearly (top insets in Figure 3C–E). The range of linearity of the Δ*D* = f(Δ*f*) dependence suggests that the antibody molecules were bound to the modified crystal surface in only one way through an amide bond. Only in the case of the process of covalent binding of anti-CD63 to the cysteamine layer was a slight deviation of the Δ*D* = f(Δ*f*) dependence from linearity observed. This observation is consistent with the conclusions obtained from the anti-CD63 surface concentration value. Probably due to the looser packing of Ab molecules in the layer, their orientation slightly deviates from the vertical.

The shape of the Δ*D* = f(Δ*f*) dependence for the step of EV interactions with the CD antibody dedicated to them strongly depends on the concentration of EVs in the analyzed solution (yellow area of the plots in the top insets in Figure 3C–E). With an increase in the EV concentration, the shape of the curve as well as the slope changed. An increase in the EV concentration results in a more densely packed layer, as evidenced by decreasing changes in the value of the dispersion coefficient. Moreover, on the Δ*D* = f(Δ*f*) dependencies obtained for the highest EV concentration (6.1 × 10^7^ particles·mL^–1^), two linear regions are well visible (top insets in Figure 3C–E, regions marked by blue lines). The first region is characterized by a smaller slope value than the second one at high degrees of saturation of the receptor layer by EVs. The presence of these two regions suggests that as the receptor layer becomes saturated by EVs, it becomes less rigid, which can be attributed to the deformation of the vesicles upon packing on the surface, ultimately leading to a more viscoelastic layer on the surface.^55^

Changing the PBS-Gibco buffer for the EV solution resulted in a further decrease in frequency (shown in Figure 3C–E, step 4). This decrease was found to be greater when a higher concentration of EVs was added to the QCM-D chamber. Calibration curves (bottom insets in Figure 3C–E) were constructed based on the changes in Δ*f* during the interaction step of EVs with the appropriate anti-CD (Figure 3C–E, step 4). A linear response was observed for the EV concentration in the range from 6.1 × 10^4^ to 6.1 × 10^7^ particles·mL^–1^ with the regression equations presented in Table 3. The LOD and LOQ values of the EV immunosensor with the QCM-D detector were determined in the same manner as in the case of the EV immunosensor with the SPR detector. To improve the QCM-D sensitivity in the EV detection, the nanostructured quartz crystal can be used.^56^

### Effect of Sample Dilution

**Figure 4:**
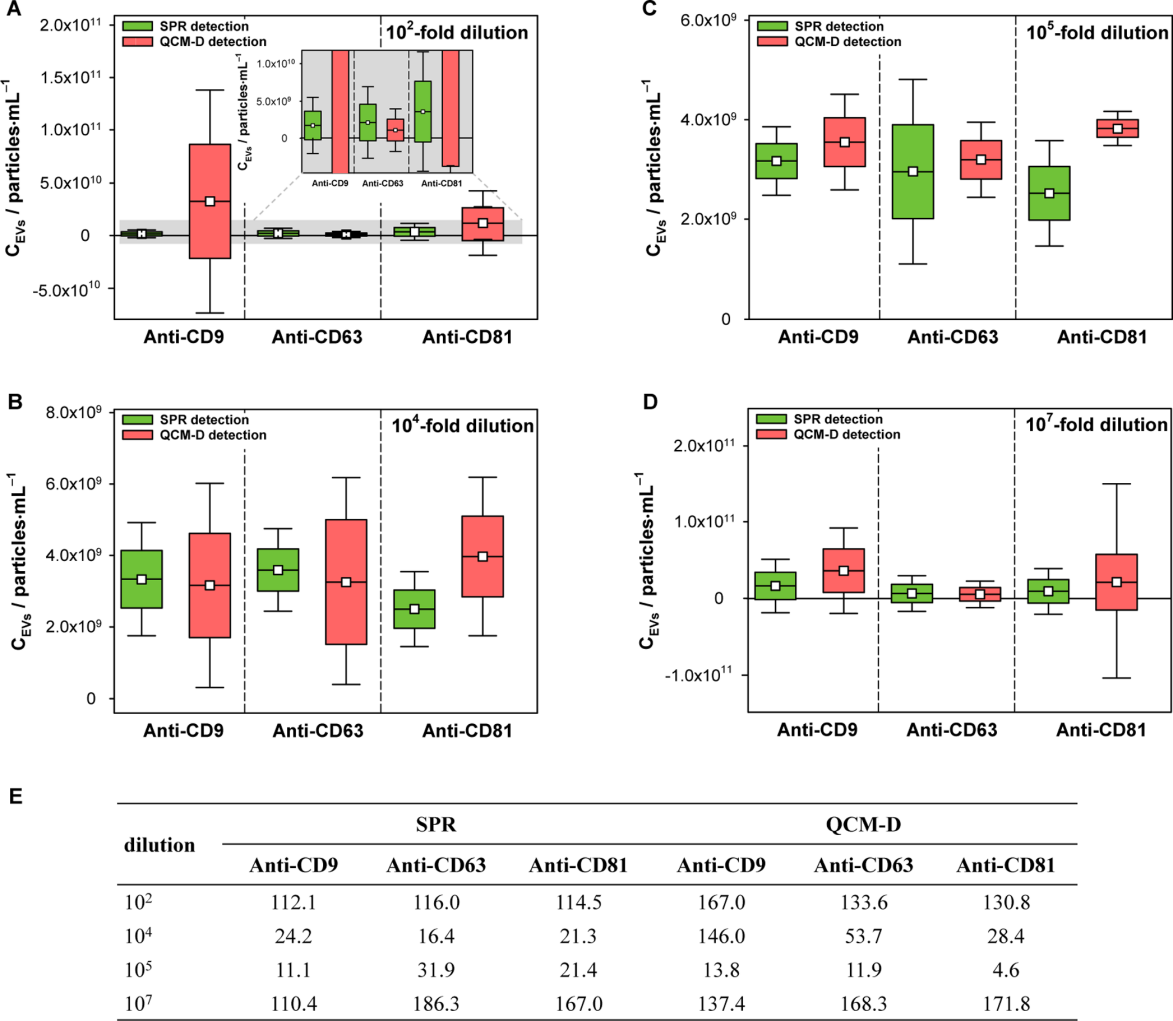
(A–D) Box plots for all tested EV solution dilutions obtained for different recognition layers using SPR and QCM-D detectors. (E) Precision (RSD %) of the method for EV analysis. Data are expressed as the mean ± SD, *n* = 3.

It is known that standard methods of EV isolation based on differential centrifugation protocols tend to induce aggregation of EVs in highly concentrated suspensions.^57^ Therefore, an important step in the study was to determine how the dilution rate of the solution affects the accuracy of the EV concentration determination using the proposed immunosensors. The study used the following dilutions: 10^2^-fold, 10^4^-fold, 10^5^-fold, and 10^7^-fold. The obtained results, presented in Figure 4, clearly demonstrate that the developed protocols work best with EV solutions diluted 10^4^-fold and 10^5^-fold for both detection techniques and all applied tetraspanin antibodies. For very concentrated (*C*_EVs_ ≥ 10^7^ particles·mL^–1^) and very diluted (*C*_EVs_ ≤ 10^4^ particles·mL^–1^) solutions, the results were unreliable. The measurement uncertainty, defined as the standard deviation, was significantly larger than the result (mean of three measurements). Validation parameters were used for two types of EV immunosensors, and the RSD (%) was determined as a measurement of precision, as shown in Figure 4E. The best statistical parameters (smallest standard deviation and very good repeatability) were obtained for measurements performed on solutions diluted 10^5^ times. Therefore, the solutions at this dilution level were used in further studies.

### Analysis of EVs Derived from Lung Cancer Cells

Due to the role of EVs in tumor metastasis, intensive studies have focused on developing increasingly sensitive methods for their detection.^58,59^ Due to the potential applications of EV analysis as biomarkers in modern medicine, there exist numerous protocols for their determination.^60^ Some of these are presented in S1 in the Supporting Information. A comparison of the proposed protocols for EV determination with those described in the literature shows that our approaches are simple, effective, and very sensitive. Although the composition of the EV membrane strictly depends on its source of origin, the membrane of each EV contains proteins important for its interaction with another cell, enabling it to enter the recipient and proteins that initiate signaling in the recipient. Specific biomarkers of the cell membrane are tetraspanins such as CD9, CD37, CD53, CD63, CD81, and CD82.^61^ Thus, the parallel analysis of several tetraspanins makes it possible to minimize errors in EV determination due to the low amount of a given biomarker in the membrane. Moreover, the proposed analytical protocols work very well in measurements carried out in the standard buffer PBS-Gibco.

It is known that each immunosensor can be regenerated using a glycine-hydrochloride solution with a pH range of 2.50–3.50.^62^ For this purpose, the sensors were exposed to a glycine-HCl solution with a pH of 2.80 for 15 min. After this time, the EVs were removed from the sensing layer, and the sensor was ready for further reuse. Four regeneration repetitions showed that the activity of the developed sensors was still at a good level, with signal intensity changes still at 90% of the initial value.

**Figure 5:**
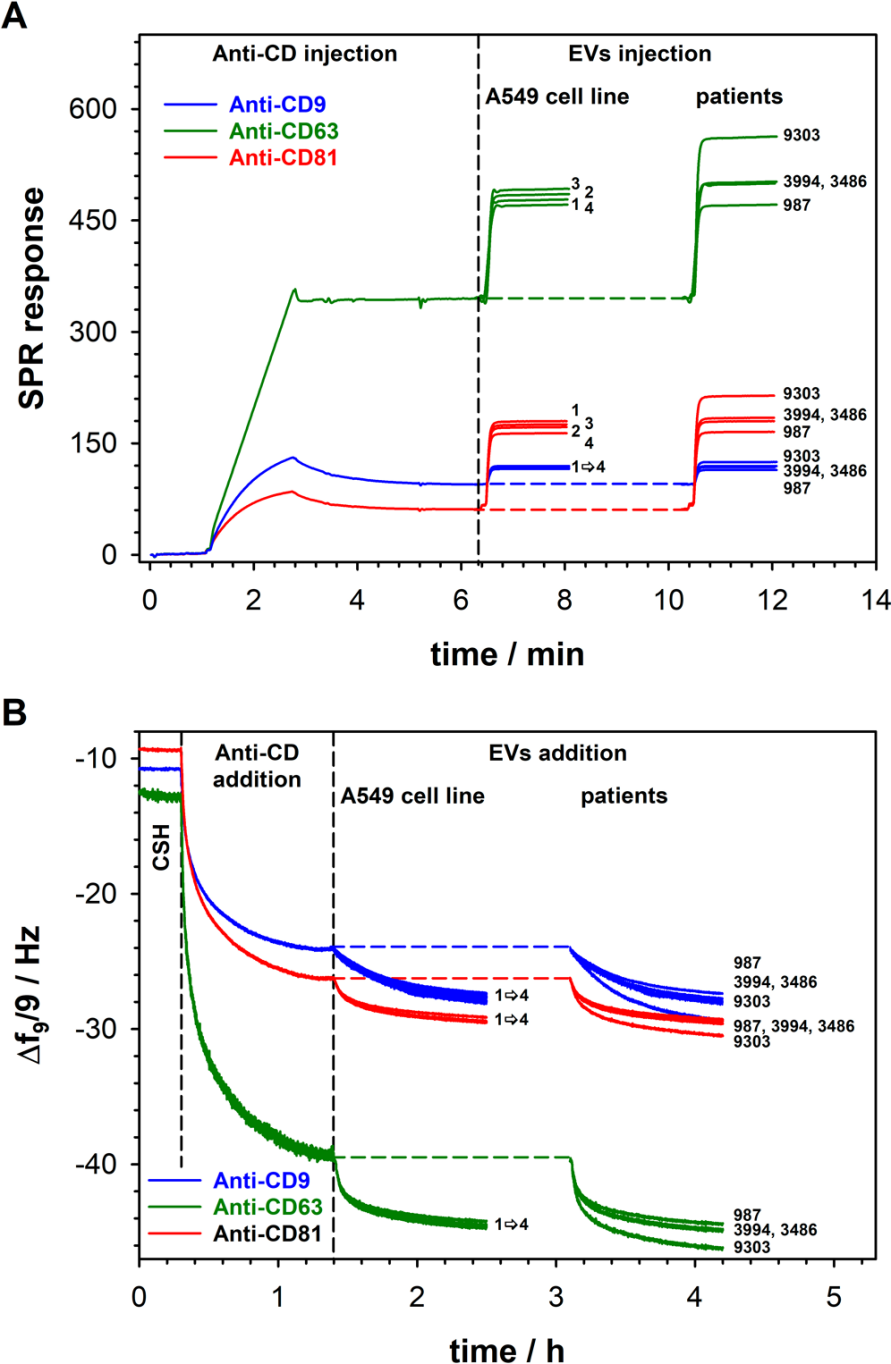
(A) Sensorgrams recorded for the interactions of anti-CD9 (black line), anti-CD63 (green line), and anti-CD81 (red line) with EVs derived from the A549 cell culture medium and patients. (B) Frequency (Δ*f*) changes recorded during the modification of the gold substrate with a cysteamine layer, appropriate antibody immobilization, and after its interaction with EVs derived from the A549 cell culture medium and patients. Experimental conditions: 0.01 M PBS-Gibco (pH 7.4; 0.005% Tween), *C*_Abs_ = 1.0 μg·mL^–1^, and *C*_EVs_: 6.1 × 10^4^ to 6.1 × 10^7^ particles·mL^–1^; dilution: 10^4^-fold.

The proposed EV labeling protocols were tested for their applicability against EVs derived from lung cancer cells (A549) and primary lung cancer cells developed based on lung cancer tissues from lung cancer patients. The results obtained are presented in Figure 5 and Table 4. A very good agreement between the results produced based on the SPR and QCM-D detectors and NTA clearly demonstrates that the developed biosensors can be successfully applied to real samples. This confirms the high potential of the proposed protocols for practical applications.

**Table 4:** Analysis of EVs Derived from Lung Cancer Cells

*C*_EVs_ × 10^–9^ [particles·mL^–1^]							
sample	NTA	SPR			QCMD		
		anti-CD9	anti-CD63	anti-CD81	anti-CD9	anti-CD63	anti-CD81
EVs Isolated from Lung Cancer Cells (A549 Cell Line)							
sample 1	3.67 ± 0.31	2.63 ± 1.05	2.97 ± 0.80	4.15 ± 1.05	4.66 ± 0.87	3.42 ± 1.03	3.45 ± 0.70
sample 2	3.23 ± 0.46	2.45 ± 0.65	3.11 ± 0.26	2.76 ± 0.62	2.89 ± 1.12	3.68 ± 0.98	4.05 ± 1.50
sample 3	3.82 ± 0.15	3.21 ± 0.75	3.45 ± 0.68	3.22 ± 0.57	3.56 ± 1.13	4.21 ± 1.12	4.30 ± 1.24
sample 4	1.80 ± 0.12	2.03 ± 0.77	2.35 ± 0.65	1.98 ± 0.35	2.07 ± 0.90	2.35 ± 1.50	2.20 ± 0.80
EVs Isolated from Lung Cancer Cells Derived from Lung Patients (Primary Cell Lines)							
987	2.60 ± 0.15	1.83 ± 0.86	2.48 ± 0.70	2.06 ± 0.78	2.26 ± 0.80	2.98 ± 1.10	3.14 ± 0.60
3486	4.22 ± 0.18	3.49 ± 1.12	4.95 ± 0.86	4.05 ± 0.87	3.98 ± 1.32	4.80 ± 1.23	3.80 ± 1.20
3994	4.78 ± 0.15	5.21 ± 0.89	4.56 ± 0.35	4.98 ± 0.46	5.40 ± 1.41	4.50 ± 1.23	5.10 ± 1.87
9303	24.3 ± 0.05	17.2 ± 2.62	26.2 ± 2.45	22.6 ± 3.75	27.9 ± 3.20	25.3 ± 2.30	27.5 ± 3.30

## Conclusions

The analysis of EVs in different body fluids and tissue samples has become a hot research topic in recent years^63^ because EVs are stable and carry diverse cargo molecules, they are considered a promising tool for noninvasive diagnosis in numerous disease states, including cancers. Characterization of EVs from body fluids can provide valuable information for early detection, disease monitoring, and the development of effective treatments against cancer.^64,65^ Cancer-derived exosomes, in particular, hold high hopes as biomarkers for early clinical diagnosis and evaluation of cancer therapeutic efficacy. However, the determination of EV concentration in solution is challenging in modern medicine due to the low concentration of tumor cell-derived EVs in body fluids and the low variety of components characteristic of such EVs. Conventional exosome detection methods are characterized by low sensitivity and reproducibility, which creates a need for new approaches for greater accuracy in size and concentration analysis. The standard methods applied in the EV analysis are NTA, tunable resistive pulse sensing, vesicle flow cytometry, and, more recently, microfluidic and other techniques that are mainly used for EV quantification.

In this study, the challenge of quantitatively analyzing EVs was addressed by combining information about the EV kinetics of attachment to an SPR sensor and the changes in mass and viscoelastic properties that occur when EVs adhere to the surfaces of piezoelectric quartz sensors. The study focused on the three tetraspanins CD9, CD63, and CD81, which are present in the membrane of EVs and can support lung cancer diagnostics. It is known that CD9 is broadly expressed in NSCLC lines but is either absent or highly reduced in most SCLC lines. On the other hand, CD63 and CD81 are broadly expressed in both SCLC and NSCLC lines.^28^ To date, most studies on QCM-D and SPR lung cancer cell-derived EVs have focused mainly on CD63 detection,^29−31^ and the analysis of only a few tetraspanins leads to the determination of only EVs in the analyzed solution, not the other EV-type particles. This study applied SPR and QCM-D to study the concentration of EVs using three well-defined tetraspanin biomarkers (CD9, CD63, and CD81) in lung cancer cells. The study used a commercially available protein A SPR sensor chip without additional modification and a QCM-D quartz gold crystal modified only with a cysteamine layer without any amplifiers. In other words, the amplifier-free SPR and QCM-D immunosensors for direct and sensitive EV quantification from lung cancer cells are a real novelty in our studies when compared with the recent literature.

## Supporting Information Available

Dependencies of ln(*R*_0_/*R*) = f(*t*) for interactions of EVs with selected monoclonal tetraspanin antibodies and exampled analytical protocols for extracellular vesicle determination (PDF)
