# Supplementary material for: Parallel SPR and QCM-D Quantitative Analysis of CD9, CD63, and CD81 Tetraspanins: A Simple and Sensitive Way to Determine the Concentration of Extracellular Vesicles Isolated from Human Lung Cancer Cells
Source: Anal Chem. 2023 Jun 12;95(25):9520–30. doi: 10.1021/acs.analchem.3c00772 (PMC10308334; doi:10.1021/acs.analchem.3c00772)
Supplement: Supplementary file 1 — ac3c00772_si_001.pdf [file ac3c00772_si_001.pdf]

SUPPORTING INFORMATION

Parallel SPR and QCM-D quantitative analysis of CD9, CD63, and CD81 tetraspanins: a simple and sensitive way to determine the concentration of extracellular vesicles isolated from human lung cancer cells

Agata Kowalczyk[1,*], Aleksandra Gajda-Walczak[1], Monika Ruzycka-Ayoush[2], Alicja Targonska[3], Grazyna Mosieniak[3], Maciej Glogowski[4], Anna Szumera-Cieckiewicz[5], Monika Prochorec-Sobieszek[5], Magdalena Bamburowicz-Klimkowska[2], Anna M. Nowicka[1,*], and Ireneusz P. Grudzinski[2]

[1]Department of Inorganic and Analytical Chemistry, Faculty of Chemistry, University of Warsaw, Pasteura Str. 1, PL-02-093, Warsaw, Poland

[2]Department of Toxicology and Food Science, Faculty of Pharmacy, Medical University of Warsaw, Banacha Str. 1, PL-02-097, Warsaw, Poland

[3]Laboratory of Molecular Bases of Ageing, Nencki Institute of Experimental Biology, Polish Academy of Sciences, Pasteura Str. 3, PL-02-093, Warsaw, Poland

[4]Department of Lung Cancer and Chest Tumors, Maria Sklodowska-Curie National Research Institute of Oncology, Roentgena Str. 5, PL-02-781, Warsaw, Poland

[5]Department of Cancer Pathomorphology, Maria Sklodowska-Curie National Research Institute of Oncology, Roentgena Str. 5, PL-02-781, Warsaw, Poland

*Corresponding authors: akowalczyk@chem.uw.edu.pl (A.K.), anowicka@chem.uw.edu.pl (A.M.N.)

A: anti-CD9

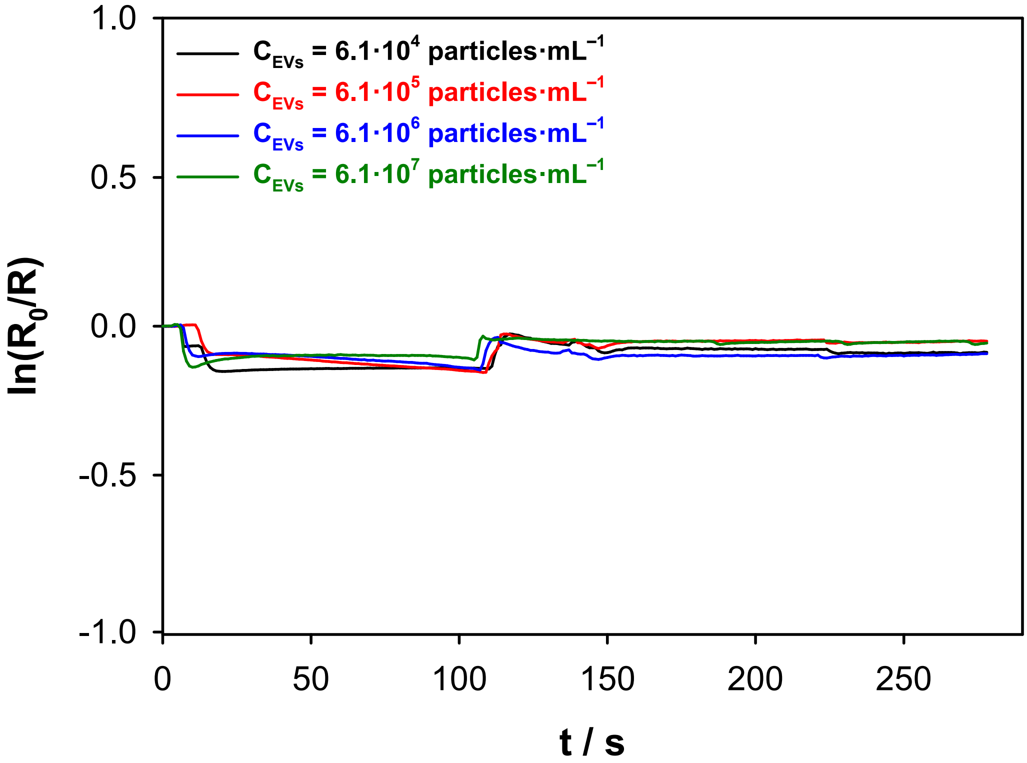

B: anti-CD63

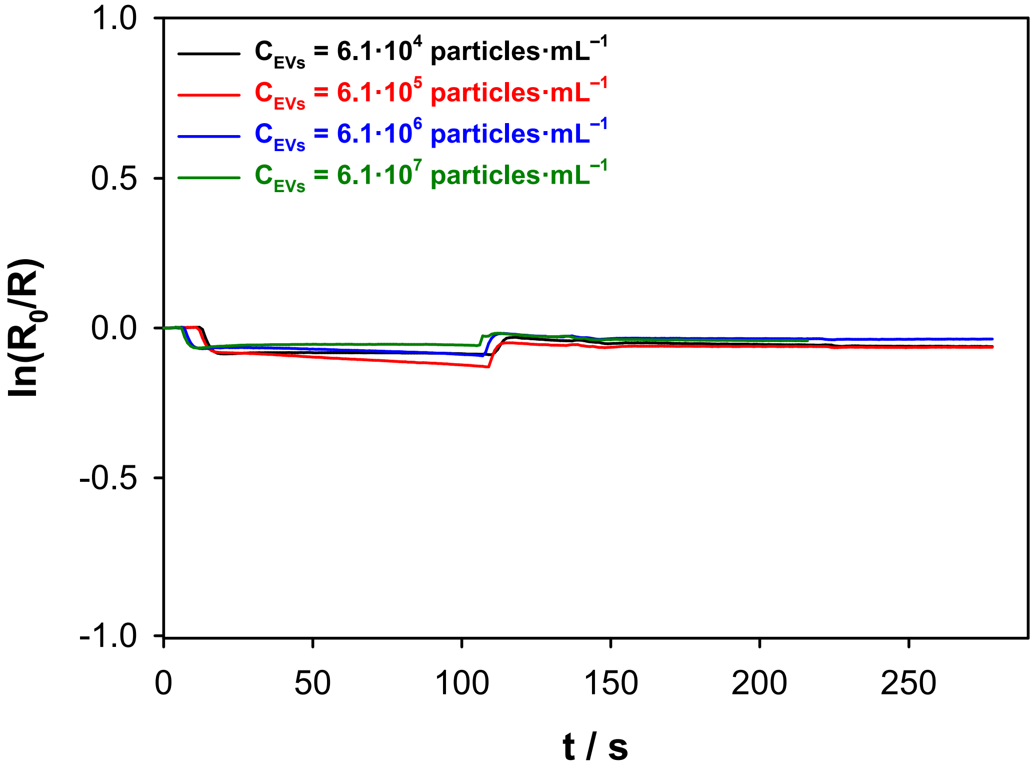

C: anti-CD81

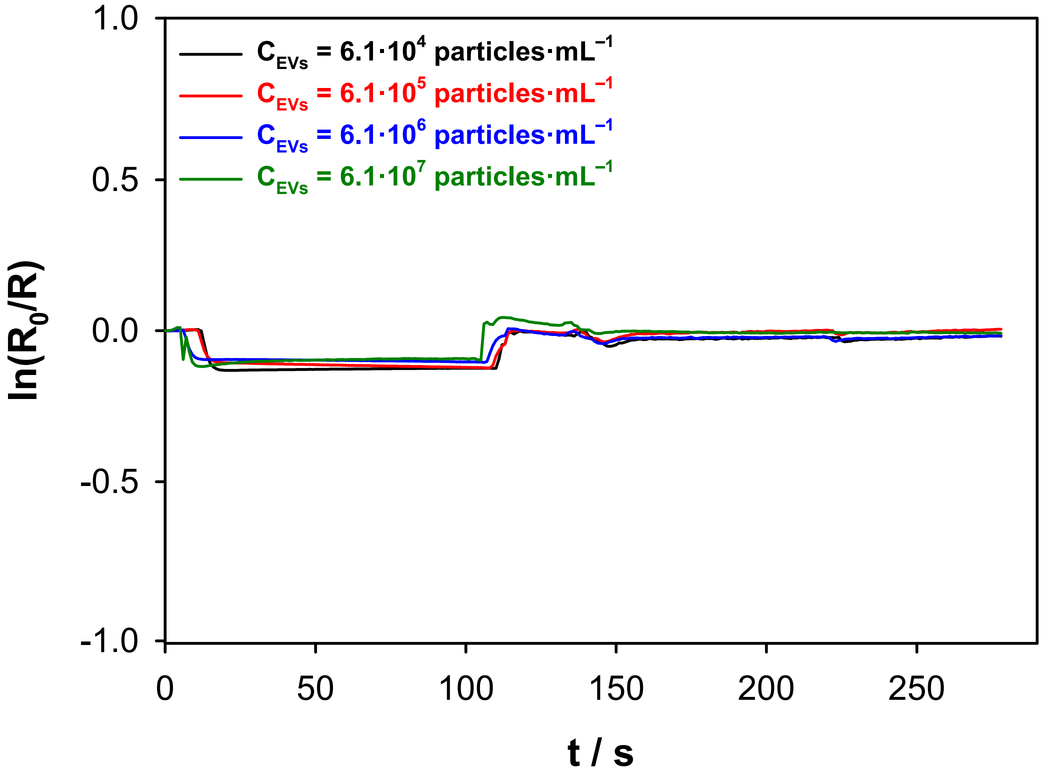

Figure S1. Dependencies of $\ln(R_0/R) = f(t)$ during interaction of extracellular vesicles with anti-CD9 (A), anti-CD63 (B) and anti-CD81 (C).

Table S1. Analytical protocols for extracellular vesicles determination.

Receptor layer	Method	Dynamic range [particles·mL^{-1}]	LOD [particles·mL^{-1}]	Ref.
Au/capture DNA/MCH/exosomes/CD 63 aptamer-T_{30}-Au NPs/A_{30}-Au NPs	dual AuNP amplified SPR aptasensor	$0 – 1·10^9$	$5·10^3$	1
Au/CD63 aptamer/DNA-MB	aptamer-based electrochemical (SWV) biosensor	$1·10^6 – 1·10^9$	$1·10^6$	2
Au/anti-EpCAM aptamers/exosomes/ Ag NPs-anti-EpCAM an Cu NPs-anti-PSMA	electrochemical (LSV) sensor with metal nanoparticles	—	$5·10^4$	3
Au/DNA nanotetranedron/aptamer LZH8	electrochemical (SWV) sensor with expanded nucleotide	$1·10^4 – 1·10^{13}$	$2.1·10^4$	4
Au/MUA/rabbit α-human CD9/exosomes/ mouse α-human CD9/ α-mouse IgG/HRP---TMB	electrochemical (ChA)sandwich immunosensor	$1·10^5 – 1·10^{11}$	$2.1·10^5$	5
Au/HS-PEG-biotin:HS-OEG-COOH/SAv/ biotin-anti-CD63	acoustic affinity immunosensor	$1·10^8 – 5·10^{10}$	$2.9·10^8$	6
Au/protein A/anti-CD9<br>Au/protein A/anti-CD63<br>Au/protein A/anti-CD81	SPR immunosensor	$6.1·10^4 – 6.1·10^7$	$7.8·10^3$<br>$0.95·10^4$<br>$2.5·10^4$	this work
Au/CSH/anti-CD9<br>Au/CSH/anti-CD63<br>Au/CSH/anti-CD81	QCM-D immunosensor	$6.1·10^4 – 6.1·10^7$	$0.60·10^4$<br>$1.8·10^4$<br>$0.70·10^4$	this work

Ag NPs: silver nanoparticles; aptamer LZH8: aptamer for HepG2 hepatocellular exosomes; Au NPs: gold nanoparticles; ChA: chronoamperommetry; CSH: cysteamine; Cu NPs: copper nanoparticles; EpCAM: epithelial cell adhesion molecule; HRP: horseradish peroxidase; LSV: linear scan voltammetry; MB: methylene blue, MCH: 6-mercapto-1-hexanol; MUA: 11-mercaptoundecanoic acid; OEG: oligo(ethylene glycol); PEG: polyethylene glycol; PSMA: prostate-specific membrane antigen; SAv: streptavidin; SPR: surface plasmon resonance; SWV: square wave voltammetry; TMB: 3,3′,5,5′-tetramethyl benzidine.

References

(1) Wang, Q.; Zou, L.; Yang, X.; Liu, X.; Nie, W.; Zheng, Y.; Cheng, Q.; Wang, K. Direct Quantification of Cancerous Exosomes via Surface Plasmon Resonance with Dual Gold Nanoparticle-Assisted Signal Amplification. *Biosens. Bioelectron.* **2019**, *135*, 129-136. https://doi.org/10.1016/j.bios.2019.04.013

(2) Zhou, Q.; Rahimian, A.; Son, K.; Shin, D. -S.; Patel, T.; Revzin, A. Development of an Aptasensor for Electrochemical Detection of Exosomes. *Methods* **2016**, *97*, 88-93. https://doi.org/10.1016/j.ymeth.2015.10.012

(3) Zhou, Y. G.; Mohamadi, R. M.; Poudineh, M.; Kermanshah, L.; Ahmed, S.; Safaei, T. S.; Stojcic, J.; Nam, R. K.; Sargent, E. H.; Kelley, S. O. Interrogating Circulating Microsomes and Exosomes Using Metal Nanoparticles. *Small* **2016**, *12*, 727-732. https://doi.org/10.1002/smll.201502365

(4) Wang, S.; Zhang, L.; Wan, S.; Cansiz, S.; Cui, C.; Liu, Y.; Wu, Y.; Dong, Y.; Tan, W. H. Aptasensor with Expanded Nucleotide Using DNA Nanotetrahedra for Electrochemical Detection of Cancerous Exosomes. *ACS Nano* **2017**, *11*, 3943-3949. https://doi.org/10.1021/acsnano.7b00373

(5) Doldán, X.; Fagúndez, P.; Cayota, A.; Laíz, J.; Tosar, J. P. Electrochemical Sandwich Immunosensor for Determination of Exosomes Based on Surface Marker-Mediated Signal Amplification. *Anal. Chem.* **2016**, *88*, 10466-10473. https://doi.org/10.1021/acs.analchem.6b02421

(6) Suthar, J.; Parsons, E. S.; Hoogenboom, B. W.; Williams, G. R.; Guldin, S. Acoustic Immunosensing of Exosomes Using a Quartz Crystal Microbalance with Dissipation Monitoring. *Anal. Chem.* **2020**, *92*, 4082-4093. https://dx.doi.org/10.1021/acs.analchem.9b05736
